# Primary Alveolar Echinococcosis of the Kidney

**DOI:** 10.1590/0037-8682-0415-2025

**Published:** 2026-02-06

**Authors:** Hicham Esselmani, Rachid Hnini, Eliane Silva, Mustapha Najimi, Mohamed Merzouki

**Affiliations:** 1 Sultan Moulay Slimane University, Faculty of Science and Technology, Biological Engineering Laboratory, Beni Mellal, Morocco.; 2 University of Porto, School of Medicine and Biomedical Sciences, Porto, Portugal.; 3 UCLouvain, Institute of Experimental and Clinical Research, Laboratory of Pediatric Hepatology and Cell Therapy, Brussels, Belgium.; 4 Private University of Marrakech, Morocco.

Alveolar echinococcosis (AE), which is caused by the larval stage of**** *Echinococcus multilocularis* **,** is a rare and aggressive parasitic disease^1^. Over 95% of cases involve the liver, with primary extrahepatic AE being exceptionally uncommon^2^. Here, we present a rare case of primary renal AE in a 67-year-old asymptomatic Moroccan man.

**FIGURE 1:**
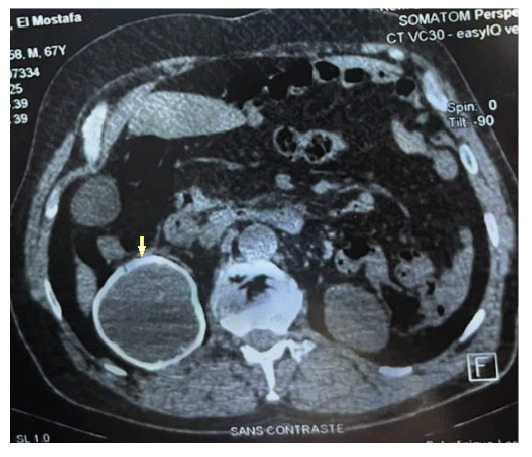
Abdominal tomography showing a calcified cyst in the right kidney with atrophy.

**FIGURE 2:**
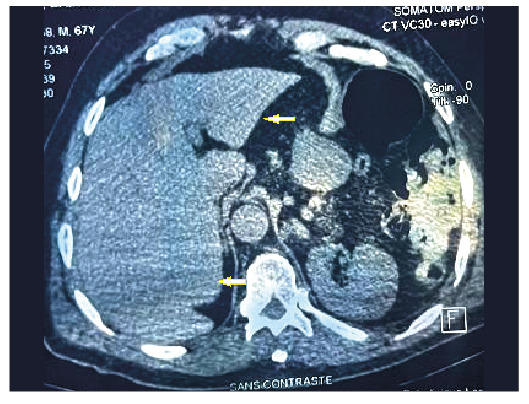
Abdominal tomography showing the liver without cystic or solid lesions.

**FIGURE 3:**
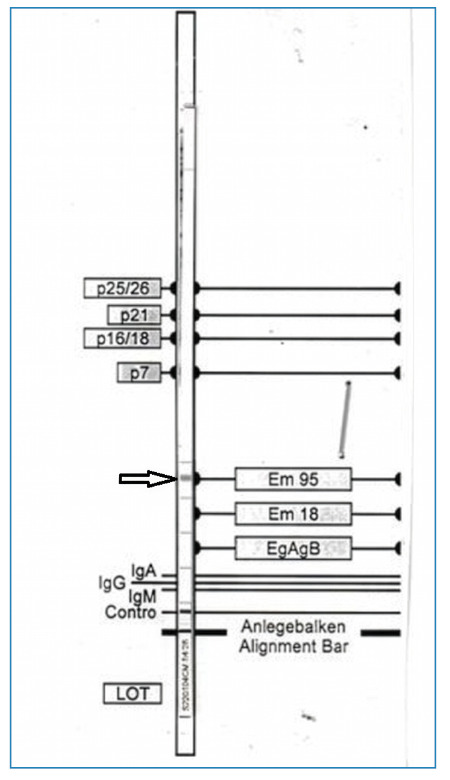
Western blot analysis showing the presence of antibodies against *E. multilocularis-*specific antigen Em95.

The lesion was incidentally detected on thoraco-abdomino-pelvic computed tomography (CT). Abdominal CT revealed a calcified cystic mass in the right kidney, associated with renal atrophy (Figure 1). The liver and other abdominal organs showed no signs of lesions (Figure 2). Serology using an indirect hemagglutination test for*E. granulosus* yielded a negative result. However, an Anti-Echinococcus EUROLINE-Western Blot IgG assay tested positive for antibodies against the*E. multilocularis*-specific antigen Em95 (Figure 3), confirming the diagnosis of AE.

To our knowledge, this represents the first case of primary renal AE reported in Morocco and the second described worldwide^3^. Isolated renal AE is a clinical mimic that can easily be misdiagnosed as a renal tumor based solely on imaging^4^. Maintaining a high index of suspicion is essential in non-endemic regions and cases with atypical presentations. Specific immunoblotting is a valuable noninvasive tool for distinguishing AE from other cystic renal lesions and for guiding appropriate management, which typically involves a combination of surgery and long-term albendazole therapy^4^.
